# Hearing Screening in Newborns: Bridging the Gap Between Normal and High-Risk Infants

**DOI:** 10.7759/cureus.107527

**Published:** 2026-04-22

**Authors:** Bhanuprakash PN, Poonam Saidha, Swetha Lakshmi M, Sai Sarath

**Affiliations:** 1 Otolaryngology, MVJ Medical College and Research Hospital, Bengaluru, IND

**Keywords:** early diagnosis, evoked potentials auditory brain stem, hearing loss/diagnosis, infant, neonatal screening, newborn, otoacoustic emissions

## Abstract

Background and objectives

Auditory perception is essential for language acquisition and social communication in early life. Undetected hearing impairment can adversely affect cognitive and developmental outcomes. Universal newborn hearing screening (UNHS) enables early identification and timely intervention. The primary objective of this study was to compare the prevalence of confirmed hearing loss between healthy and high-risk neonates. Secondary objectives included evaluating the effectiveness of sequential screening using otoacoustic emissions (OAE) followed by brainstem evoked response audiometry (BERA), assessing referral rates at different stages of the screening protocol, and identifying risk factors associated with confirmed hearing loss among high-risk neonates.

Methods

This prospective observational study was conducted over a period of 12 months at a tertiary care center, including 1,460 neonates: 945 classified as healthy and 515 as high-risk based on established clinical criteria. All infants underwent initial OAE testing within 48 hours of birth. Those who did not pass were retested after two weeks. Infants who failed both screenings underwent BERA within three months for confirmation.

Results

Among 945 healthy neonates, 87 failed the initial OAE, with 13 remaining as referral cases after repeat testing. In the high-risk group (n = 515), 147 failed the first OAE, and 36 continued to fail after repeat testing. BERA confirmed hearing impairment in 17 infants, predominantly in the high-risk group. The prevalence of confirmed hearing loss was 5.3 per 1,000 in healthy neonates and 23.3 per 1,000 in high-risk neonates.

Conclusions

A significantly higher prevalence of confirmed hearing loss was observed among high-risk neonates compared to healthy infants. However, the presence of hearing impairment in infants without identifiable risk factors supports the need for UNHS. Sequential screening using OAE followed by confirmatory BERA is effective for early and accurate diagnosis.

## Introduction

Auditory input from infancy is crucial for acquiring language, forming social bonds, and enabling effective communication. If left undetected, hearing loss can hinder academic progress, social adjustment, and emotional well-being. Universal newborn hearing screening (UNHS) initiatives aim to identify auditory deficits at the earliest possible stage to enable timely intervention and minimize developmental delays. The auditory system undergoes critical development during fetal life, with key structural and neural maturation continuing into early infancy, particularly within the first 12 weeks after birth [1]. Hearing loss remains a global concern, with a significant prevalence across age groups [2].

Early identification of infants at risk enables their enrollment in early intervention programs, ideally by six months of age, which helps reduce language and cognitive deficits associated with delayed diagnosis [3,4]. According to the 2007 guidelines published by the Joint Committee on Infant Hearing (JCIH), all newborns should undergo hearing screening within the first month of life, followed by a confirmatory assessment by three months and appropriate intervention by six months [5]. While high-risk neonates have a greater probability of hearing impairment, a considerable proportion of affected infants do not exhibit identifiable risk factors. This highlights the importance of universal screening and emphasizes the need for a clearer understanding of the distribution of hearing loss among both low-risk and high-risk groups [6].

Despite widespread implementation of universal newborn hearing screening programs, existing literature demonstrates considerable variability in the reported prevalence of neonatal hearing loss across different populations. While several studies report a significantly higher prevalence among high-risk neonates, others have shown that a substantial proportion of affected infants do not present with identifiable risk factors, thereby questioning the effectiveness of selective screening strategies. Additionally, variations in reported prevalence may be influenced by differences in screening methodologies, timing of screening, population characteristics, and follow-up compliance.

In developing countries such as India, the implementation of universal newborn hearing screening presents additional challenges, including variability in healthcare infrastructure, limited access to diagnostic facilities, and inconsistent follow-up. Reported prevalence rates in similar settings range approximately from one to six per 1,000 live births in the general neonatal population, with substantially higher rates among high-risk infants [4]. These variations highlight the need for region-specific data to better understand the burden of confirmed hearing impairment and to optimize screening strategies within local healthcare systems.

The primary screening tools - otoacoustic emissions (OAE) and brainstem evoked response audiometry (BERA) - provide reliable assessment of cochlear and neural auditory function, respectively [7]. This study aims to compare the prevalence of hearing loss between normal and high-risk neonates using a structured universal newborn hearing screening protocol. Secondary objectives included evaluating the effectiveness of sequential screening using OAE followed by BERA, assessing referral rates at different stages of the screening protocol, and identifying risk factors associated with confirmed hearing loss among high-risk neonates. We hypothesize that high-risk neonates will demonstrate a higher prevalence of hearing impairment compared to normal neonates; however, a clinically significant proportion of hearing loss will also be detected among infants without identifiable risk factors, thereby supporting the need for universal newborn hearing screening.

## Materials and methods

This prospective, non-randomized observational study was conducted in the Department of ENT at a tertiary care teaching hospital from March 2024 to February 2025. A total of 1,460 neonates born during the study period were included after obtaining written informed consent from parents or legal guardians. All live-born neonates delivered at the study center during the study period, whose parents provided informed consent, were included in the study. Neonates with external ear anomalies precluding OAE testing, those with incomplete clinical data, neonates whose parents did not consent to participate, and those lost to follow-up before confirmatory BERA testing were excluded.

Participants were categorized into normal and high-risk groups based on clinical history and predefined risk criteria. A detailed perinatal history, including prenatal, natal, and postnatal factors, was recorded. High-risk neonates were identified according to the JCIH 2019 guidelines [5], including prematurity (<34 weeks), low birth weight (<1.5 kg), birth asphyxia, neonatal hyperbilirubinemia, congenital infections, craniofacial anomalies, ototoxic drug exposure, bacterial meningitis, low Apgar scores, and prolonged mechanical ventilation (>5 days).

All neonates underwent initial hearing screening using Otoacoustic Emissions (OAE) within 48 hours of birth using a Maico ERO•SCAN handheld screener (MAICO Diagnostics GmbH, Berlin, Germany). Infants who failed the second OAE screening were subjected to BERA using the Labat Epic Plus system (Labat Asia Pvt. Ltd., Mohali, India). All OAE and BERA assessments were performed using standardized protocols with regularly calibrated equipment. Testing was conducted by trained personnel to ensure consistency and minimize inter-operator variability.

Outcome measures

The primary outcome was the prevalence of confirmed hearing loss among normal and high-risk neonates. Secondary outcomes included referral rates following initial and repeat OAE screening, effectiveness of the sequential screening protocol, and identification of risk factors associated with confirmed hearing loss. Confirmed hearing loss was defined as abnormal findings on BERA in infants who failed both initial and repeat OAE screening.

Sample size calculation

The sample size was calculated based on the expected prevalence of hearing loss among neonates using the standard formula for the estimation of a proportion:

$n=\frac{Z^{2}p(1-p)}{d^{2}}$ [8]

where n is the required sample size, Z is the standard normal deviate corresponding to a 95% confidence level (CI) (1.96), p is the expected prevalence of hearing loss, and d is the allowable error.

Based on previous studies, the prevalence of neonatal hearing loss was estimated at approximately 2% (p = 0.02). With an absolute precision of 1% (d = 0.01) and 95% CI, the minimum calculated sample size was approximately 753. Considering possible attrition and loss to follow-up, the sample size was increased, and all eligible neonates during the study period were included, resulting in a total sample of 1,460 participants.

Ethical consideration

The study was approved by the Institutional Ethics Committee of MVJ Medical College & Research Hospital, Bangalore (approval no: MVJMC&RH/IEC-130/2024; date: 14-02-2024). The study was conducted in accordance with the principles of the Declaration of Helsinki. Written informed consent was obtained from the parents or legal guardians of all participants before inclusion in the study.

Statistical analysis

Data were analyzed using IBM SPSS Statistics version 26.0 (IBM Corp., Armonk, NY). Categorical variables were expressed as frequencies and percentages, while continuous variables were expressed as mean ± standard deviation (SD). The Chi-square test was used to compare proportions between groups, and the independent samples t-test was used to compare continuous variables. Multivariate logistic regression analysis was performed to identify independent predictors of hearing loss among high-risk neonates. Adjusted odds ratios (aORs) with 95% CIs were calculated. A p-value < 0.05 was considered statistically significant.

## Results

**Figure 1:**
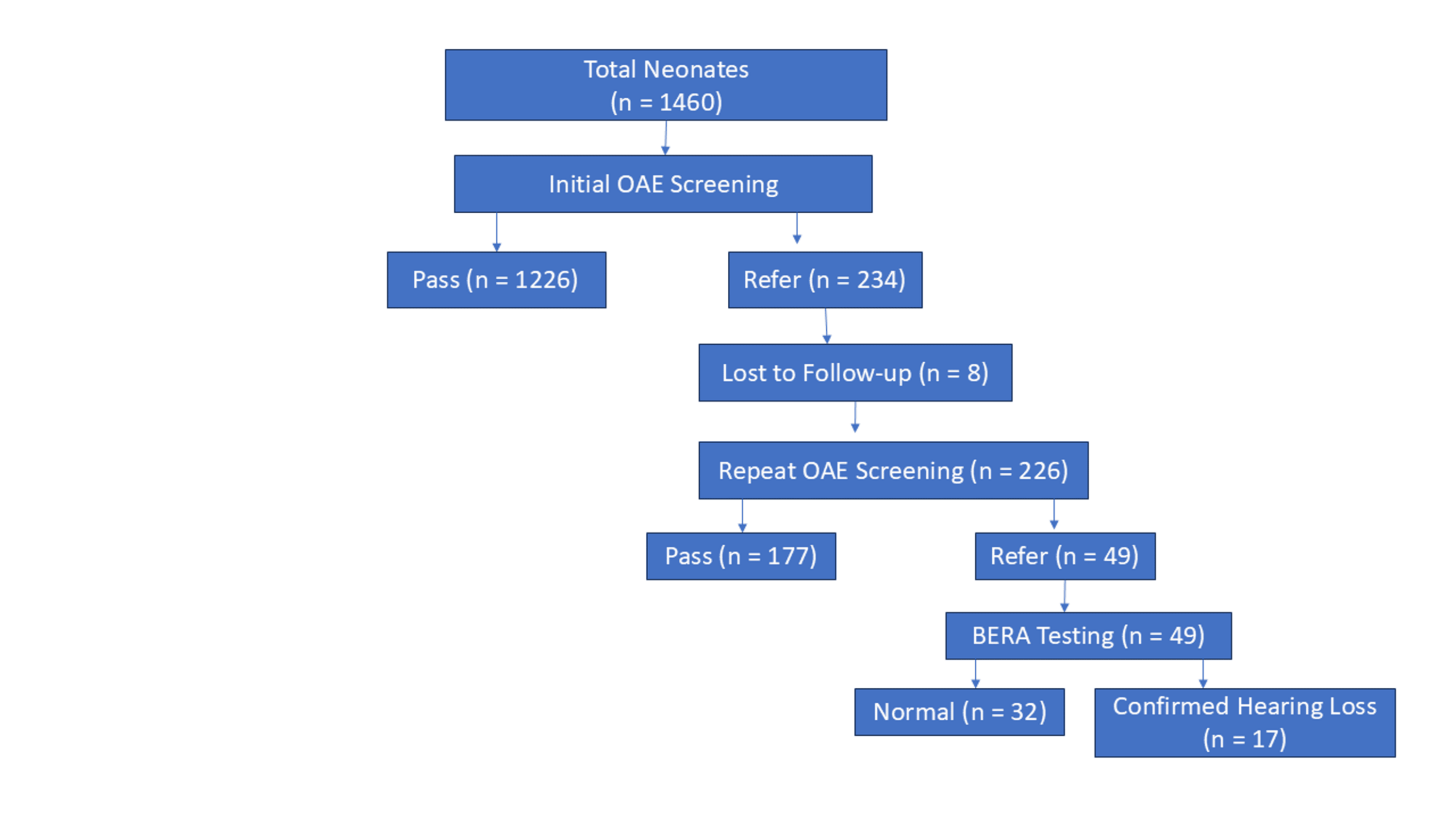
Flowchart of newborn hearing screening protocol and outcomes Flowchart showing the sequential newborn hearing screening pathway using OAE followed by BERA, with final outcomes categorized as normal hearing or confirmed hearing loss OAE: otoacoustic emissions; BERA: brainstem evoked response audiometry

The flow of participants through the sequential newborn hearing screening protocol is illustrated in Figure 1. A total of 1,460 neonates underwent initial OAE screening, of whom the majority passed while a subset was referred for repeat testing. After excluding those lost to follow-up, infants who failed the initial screening underwent repeat OAE, resulting in a further reduction in referral cases. Infants who failed both screening stages were subjected to confirmatory BERA testing, through which a proportion of cases were diagnosed with confirmed hearing loss.

The baseline characteristics of the study population are summarized in Table 1. A total of 1,460 neonates were included, comprising 945 (64.7%) normal and 515 (35.3%) high-risk infants. Gender distribution was comparable between the groups (p = 0.09). High-risk neonates had significantly lower gestational age and birth weight compared to normal neonates (p < 0.001), and a higher proportion were delivered by cesarean section (p < 0.001).

**Table 1:** Baseline characteristics of the study population ^*^Statistically significant Chi-square (χ²) test was used for categorical variables, and an independent t-test was used for continuous variables. A p-value < 0.05 was considered statistically significant SD: standard deviation

Characteristic	Normal neonates (n = 945)	High-risk neonates (n = 515)	Test statistic	P-value
Gender, n (%)			χ² = 2.86	0.09
Male	510 (54.0%)	301 (58.4%)	—	—
Female	435 (46.0%)	214 (41.6%)	—	—
Gestational age			t = 24.5	< 0.001^*^
Mean ± SD (weeks)	39 ± 1.2	36 ± 2.4	—	—
Preterm (<37 weeks), n (%)	0 (0%)	76 (14.8%)	—	—
Birth weight			t = 41.2	< 0.001^*^
Mean ± SD (kg)	3.0 ± 0.5	1.9 ± 0.4	—	—
Low birth weight (<2.5 kg), n (%)	0 (0%)	83 (16.1%)	—	—
Mode of delivery, n (%)			χ² = 290.3	< 0.001^*^
Normal vaginal delivery	774 (81.9%)	196 (38.1%)	—	—
Cesarean section	171 (18.1%)	319 (61.9%)	—	—

Initial OAE screening

Following the initial OAE screening, the overall pass rate was higher among normal neonates, while referral rates were significantly greater in the high-risk group (p < 0.0001) (Table 2).

**Table 2:** Initial OAE screening outcomes by infant group ^*^Statistically significant Chi-square (χ²) test was used for comparison between groups. A p-value < 0.05 was considered statistically significant OAE: otoacoustic emissions

Group	Passed, n (%)	Referral, n (%)	Test statistic	P-value
Normal (n = 945)	858 (90.8%)	87 (9.2%)	—	—
High-risk (n = 515)	368 (71.5%)	147 (28.5%)	—	—
Total (n = 1460)	1226 (84.0%)	234 (16.0%)	χ² = 104.2	< 0.0001^*^

Among infants who failed the initial screening, most underwent repeat testing after excluding those lost to follow-up. Bilateral involvement was the most common pattern of failure, with comparable distribution between groups (Table 3).

**Table 3:** Laterality of initial OAE failures (follow-up cases) Chi-square (χ²) test was used for comparison between groups. A p-value < 0.05 was considered statistically significant OAE: otoacoustic emissions

Group	Right ear, n (%)	Left ear, n (%)	Both ears, n (%)	Total	Test statistic	P-value
Normal (n = 82)	24 (29.3%)	21 (25.6%)	37 (45.1%)	82	—	—
High-risk (n = 144)	33 (22.9%)	43 (29.9%)	68 (47.2%)	144	—	—
Total (n = 226)	57 (25.2%)	64 (28.3%)	105 (46.5%)	226	χ² = 1.21	0.54

Repeat OAE screening

After repeat screening, the majority of infants passed, with no statistically significant difference between normal and high-risk groups (p = 0.12) (Table 4).

**Table 4:** Repeat OAE screening outcomes Chi-square (χ²) test was used for comparison between groups. A p-value < 0.05 was considered statistically significant OAE: otoacoustic emissions

Group	Passed, n (%)	Referral, n (%)	Total	Test statistic	P-value
Normal (n = 82)	69 (84.1%)	13 (15.9%)	82	—	—
High-risk (n = 144)	108 (75.0%)	36 (25.0%)	144	—	—
Total (n = 226)	177 (78.3%)	49 (21.7%)	226	χ² = 2.41	0.12

BERA confirmation

Among infants who failed both OAE screenings, a proportion were confirmed to have hearing loss on BERA, with no statistically significant difference between normal and high-risk groups (p = 0.73) (Table 5).

**Table 5:** Diagnostic confirmation using BERA among infants referred after repeat OAE Chi-square (χ²) test was used for comparison between groups. A p-value < 0.05 was considered statistically significant BERA: brainstem evoked response audiometry; OAE: otoacoustic emissions

Group	Hearing loss, n (%)	No hearing loss, n (%)	Total	Test statistic	P-value
Normal (n = 13)	5 (38.5%)	8 (61.5%)	13	—	—
High-risk (n = 36)	12 (33.3%)	24 (66.7%)	36	—	—
Total (n = 49)	17 (34.7%)	32 (65.3%)	49	χ² = 0.12	0.73

Prevalence of hearing loss

Among the total study population, 17 infants were diagnosed with confirmed hearing loss, corresponding to an overall prevalence of 11.6 per 1,000 live births. The prevalence was significantly higher in high-risk neonates compared to normal neonates (23.3 vs 5.3 per 1,000; p = 0.002) (Table 6).

**Table 6:** Prevalence of hearing loss among the entire study population ^*^Statistically significant Values are expressed as a number or prevalence per 1,000 live births. Chi-square (χ²) test was used for comparison between groups. A p-value < 0.05 was considered statistically significant

Group	Hearing loss	No hearing loss	Total	Prevalence (/1000)	Test statistic	P-value
Normal	5	940	945	5.3	—	—
High-risk	12	503	515	23.3	—	—
Total	17	1443	1460	NA	χ² = 9.61	0.002^*^

**Figure 2:**
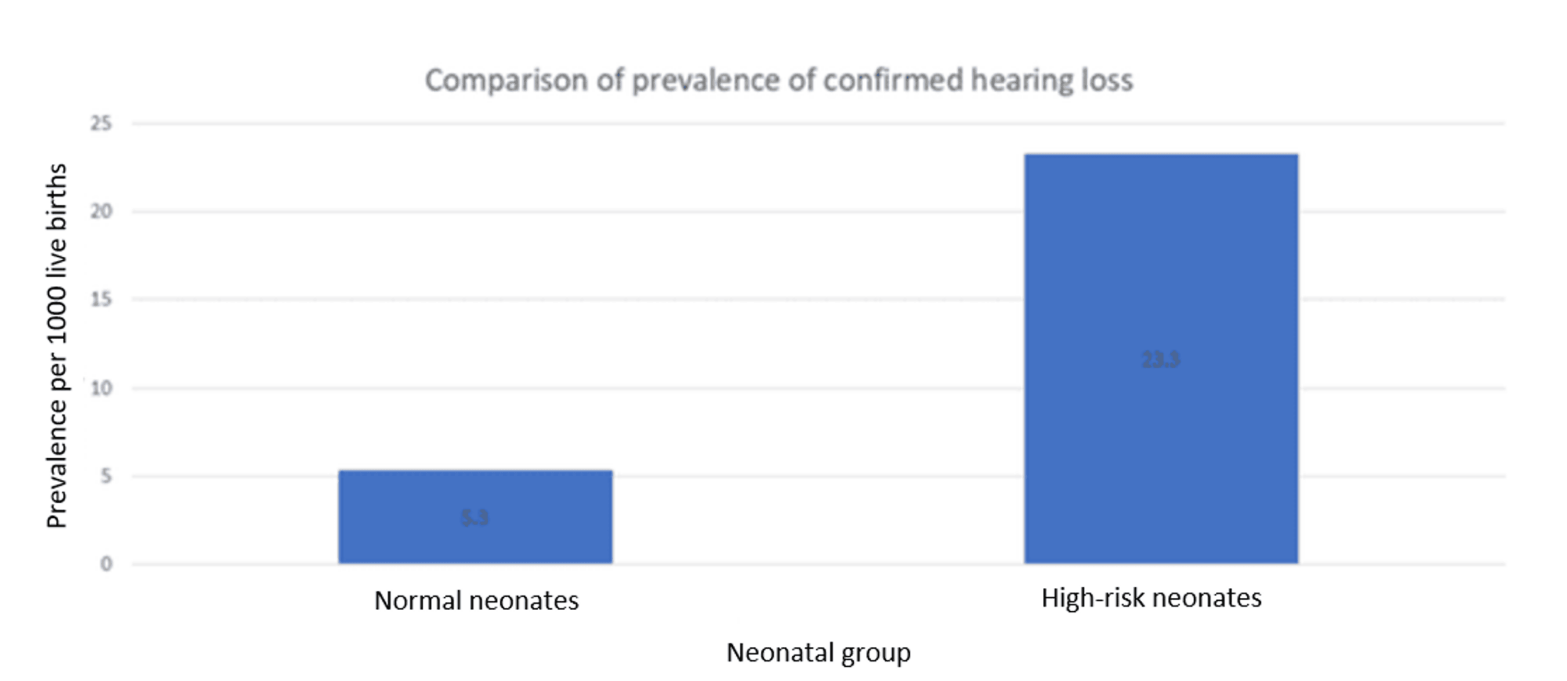
Comparison of prevalence of confirmed hearing loss between normal and high-risk neonates Bar chart showing higher prevalence of confirmed hearing loss among high-risk neonates compared to normal neonates

The prevalence of confirmed hearing loss was significantly higher among high-risk neonates compared to normal neonates (23.3 vs. 5.3 per 1,000; p = 0.002), as shown in Table 6 and illustrated in Figure 2.

Risk factor analysis

Within the high-risk group, several perinatal factors were associated with confirmed hearing loss, including craniofacial anomalies, consanguinity, birth asphyxia, meconium aspiration, and meningitis (Table 7).

**Table 7:** Distribution of risk factors among high-risk neonates and hearing loss outcomes This table presents the distribution of risk factors among high-risk neonates and the corresponding hearing outcomes OAE: otoacoustic emissions; BERA: brainstem evoked response audiometry; NICU: neonatal intensive care unit

Risk factor	Total (n = 515)	Initial OAE referral (n = 147)	Repeat OAE referral (n = 36)	Hearing loss (BERA), n (%)
Prenatal factors	—	—	—	—
Genetic history of hearing loss	58	12	3	1 (8.3%)
Parental consanguinity	45	14	5	2 (16.7%)
Exposure to ototoxic drugs	5	3	1	0 (0%)
Maternal infections	8	0	0	0 (0%)
Natal factors	—	—	—	—
Prematurity (< 37 weeks)	76	24	3	1 (8.3%)
Low birth weight (< 2.5 kg)	83	18	2	0 (0%)
Birth asphyxia	24	9	4	2 (16.7%)
Meconium aspiration	17	6	0	2 (16.7%)
Neonatal jaundice	47	12	2	0 (0%)
Meningitis	15	7	3	2 (16.7%)
NICU admission (> 5 days)	57	15	3	1 (8.3%)
Postnatal factors	—	—	—	—
Craniofacial anomalies	4	2	1	1 (25.0%)
Seizures/convulsions	28	14	5	1 (8.3%)
Recurrent ear infections	48	11	4	1 (8.3%)

Multivariate logistic regression analysis identified parental consanguinity, birth asphyxia, meconium aspiration, meningitis, and craniofacial anomalies as significant independent predictors of confirmed hearing loss. Other factors were not significantly associated (Table 8).

**Table 8:** Multivariate logistical regression analysis of risk factors associated with hearing loss in high-risk neonates ^*^Statistically significant Multivariate logistic regression analysis was performed to identify independent predictors of hearing loss. A p-value < 0.05 was considered statistically significant aOR: adjusted odds ratio; CI: confidence interval; NICU: neonatal intensive care unit

Risk factor	aOR	95% CI	P-value
Genetic history of hearing loss	1.34	0.12 – 15.18	0.8
Parental consanguinity	4.65	1.01 – 21.48	0.048^*^
Prematurity	1.12	0.10 – 11.91	0.92
Birth asphyxia	5.83	1.06 – 32.01	0.043^*^
Meconium aspiration	7.15	1.15 – 44.24	0.035^*^
Meningitis	6.41	1.03 – 39.97	0.046^*^
NICU admission (>5 days)	1.17	0.14 – 9.69	0.88
Craniofacial anomalies	13.25	1.06 – 165.38	0.045^*^
Seizures/convulsions	1.07	0.13 – 8.82	0.95
Recurrent ear infections	1.33	0.16 – 10.94	0.78

## Discussion

Early detection of hearing impairment is essential for timely intervention to support speech, language, and cognitive development in children [9,10]. The present study aimed to compare the prevalence of confirmed hearing loss between healthy and high-risk neonates using a structured newborn hearing screening protocol. Our findings demonstrated a statistically significantly higher prevalence of confirmed hearing loss among high-risk neonates compared to those without identifiable risk factors. This is consistent with previous studies, which have shown that infants with perinatal complications are at increased risk for auditory impairment [5]. The higher referral rates observed in high-risk infants during initial OAE screening further reflect the influence of factors such as prematurity, low birth weight, neonatal hyperbilirubinemia, and infections [11,12].

Importantly, a proportion of infants without identifiable risk factors also exhibited hearing impairment. This finding reinforces the need for universal newborn hearing screening, as selective screening based solely on risk factors may fail to identify a substantial number of affected infants. Previous reports suggest that up to 50% of newborns with hearing loss may not present with known risk indicators [13,14].

The prevalence of confirmed hearing loss observed in our study (5.3 per 1,000 in normal infants and 23.3 per 1,000 in high-risk infants) is comparable to findings reported in other studies, although variations exist. Upadhyay et al. reported prevalence rates of 2.9 per 1,000 in normal infants and 41.38 per 1,000 in high-risk neonates [15], while Vaid et al. observed lower rates of 1.62 and 7.95 per 1,000, respectively [16]. Similarly, Ravi et al. and Parida et al. documented higher prevalence among high-risk infants compared to low-risk groups [17,18]. Such variability may be attributed to differences in study design, screening protocols, population characteristics, and follow-up rates. Paul et al. reported comparatively lower prevalence rates in both groups, further highlighting regional and methodological differences [19].

Analysis of risk factors in the present study identified parental consanguinity, birth asphyxia, meningitis, meconium aspiration, and craniofacial anomalies, which can affect cochlear and neural auditory pathways, as significant predictors of hearing loss. These findings align with previous studies that have emphasized the role of perinatal and structural factors in the development of auditory impairment [20,21]. In contrast, the lack of statistically significant association between certain expected risk factors, such as prematurity and NICU stay, and confirmed hearing loss in this study may be attributed to the relatively small number of outcome events, which limits statistical power. Additionally, variability in the severity of these conditions and differences in clinical management may influence their impact on auditory outcomes. These findings highlight the need for cautious interpretation and further investigation in larger cohorts.

The relatively high referral rate following initial OAE screening may reflect a higher false-positive rate, which is commonly observed in early neonatal screening due to transient factors such as vernix caseosa, debris in the external auditory canal, or middle ear fluid [22]. The significant reduction in referral rates after repeat screening in our study highlights the effectiveness of a two-stage screening protocol in minimizing false-positive results and avoiding unnecessary parental anxiety and additional testing. Loss to follow-up remains a significant challenge in newborn hearing screening programs. Factors such as lack of parental awareness, socioeconomic barriers, and accessibility issues can impact adherence to follow-up protocols. Strengthening parental counseling and improving accessibility to diagnostic services are essential to enhance program effectiveness.

An important and somewhat unexpected finding in the present study was the detection of confirmed hearing loss among neonates without identifiable risk factors. Subclinical or unrecognized risk factors, genetic predispositions, or environmental influences may contribute to hearing impairment in infants classified as normal. These findings highlight the limitations of risk-based screening and further support the implementation of universal screening approaches to ensure that affected infants are not missed.

The strengths of this study include a large sample size and the use of a structured sequential screening protocol combining OAE and confirmatory BERA. However, as this was a non-randomized, single-center observational study, there is a potential risk of selection bias and limited generalizability. Additionally, the relatively small number of confirmed hearing loss cases may limit statistical power and contribute to wider confidence intervals. Blinding of assessors was not performed, which may introduce measurement bias. Although standardized protocols and calibrated equipment were used, some degree of operator-dependent variability cannot be completely excluded. A small proportion of infants were lost to follow-up, and their exclusion may introduce a potential risk of attrition bias. Although the number was limited, their exclusion may have a minor impact on the estimated prevalence and outcome measures. However, given the small proportion relative to the total sample size, the overall effect on study findings is likely minimal. The absence of a statistically significant difference in BERA-confirmed hearing loss between groups may be attributed to the limited number of confirmed cases. Therefore, the findings should be interpreted with caution.

The present study contributes to the existing literature by providing a direct comparison of hearing loss prevalence between normal and high-risk neonates within a structured universal screening framework. It also demonstrates the effectiveness of a sequential OAE-BERA protocol in reducing false-positive referrals in a real-world clinical setting. Furthermore, the identification of significant risk factors such as craniofacial anomalies, consanguinity, and perinatal complications adds to the understanding of predictors of neonatal hearing loss in similar healthcare settings. From a clinical and public health perspective, the findings of this study support the implementation of universal newborn hearing screening programs rather than selective screening based solely on risk factors. Additionally, enhanced surveillance and follow-up of high-risk neonates, particularly those with identified risk factors, may facilitate early detection and intervention.

## Conclusions

This study demonstrates a significantly higher prevalence of confirmed hearing loss among high-risk neonates compared to healthy infants, while also identifying affected cases among infants without identifiable risk factors, thereby reinforcing the need for universal newborn hearing screening. The sequential OAE-BERA protocol was effective in reducing false-positive referrals and facilitating early diagnosis. Additionally, the identification of key risk factors provides clinically relevant insights for targeted monitoring and follow-up. These findings support the integration of structured universal newborn hearing screening into routine neonatal care and highlight the importance of strengthening follow-up and early intervention services, particularly in resource-limited settings.
